# Interaction Between LncRNA and UPF1 in Tumors

**DOI:** 10.3389/fgene.2021.624905

**Published:** 2021-03-01

**Authors:** Junjian He, Xiaoxin Ma

**Affiliations:** ^1^Department of Obstetrics and Gynecology, Shengjing Hospital of China Medical University, Shenyang, China; ^2^Key Laboratory of Maternal-Fetal Medicine of Liaoning Province, Key Laboratory of Obstetrics and Gynecology of Higher Education of Liaoning Province, Shenyang, China

**Keywords:** LncRNA – long non-coding RNA, UPF1, RBP, tumor, post-transcriptional regulation of gene expression

## Abstract

Long non-coding RNAs (LncRNAs) can bind to other proteins or RNAs to regulate gene expression, and its role in tumors has been extensively studied. A common RNA binding protein, UPF1, is also a key factor in a variety of RNA decay pathways. RNA decay pathways serve to control levels of particular RNA molecules. The expression of UPF1 is often dysregulated in tumors, an observation which suggests that UPF1 contributes to development of a variety of tumors. Herein, we review evidence from studies of fourteen lncRNAs interact with UPF1. The interaction between lncRNA and UPFI provide fundamental basis for cell transformation and tumorigenic growth.

## Introduction

**FIGURE 1:**
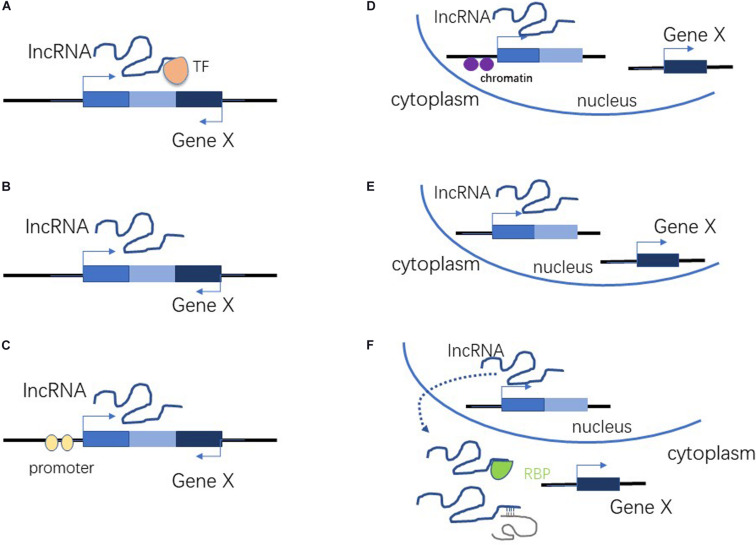
*Cis-*acting and *trans-*acting roles of lncRNA. **(A)** LncRNA recruits transcription factors to a locus to regulate the expression of nearby genes. **(B)** Regulation of nearby genes during lncRNA transcription and shearing. **(C)** Regulation of nearby genes by lncRNA promoter or original DNA of the locus. **(D)** Regulation of chromatin state and gene transcription in distant regions. **(E)** Regulation of nuclear structure and organization. **(F)** Binding to proteins or other RNAs to regulate their activity.

**FIGURE 2:**
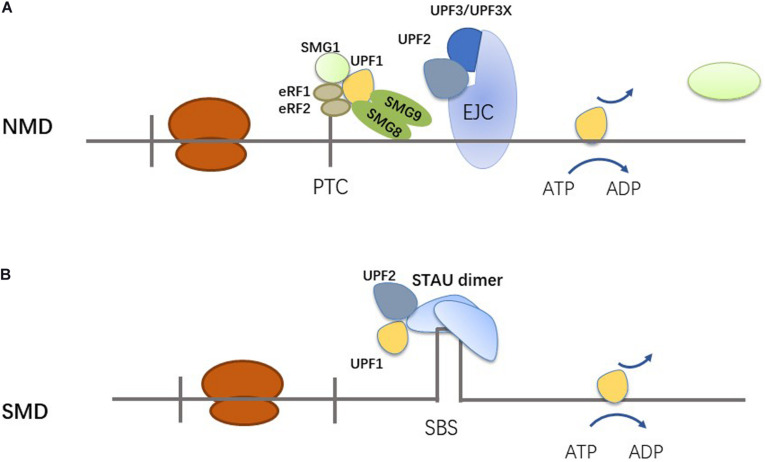
Mechanisms of NMD and SMD. **(A)** UPF1 is engaged in NMD via binding PTC through the translation termination complex. **(B)** UPF1 is engaged in the SMD via 3′ UTR-bound STAU1 or STAU2.

About 93% of the human genome is transcribed into RNA. Protein-coding genes account for only about 2% of RNAs, with the vast majority of transcripts being non-coding RNAs (ncRNAs) (Qian et al., 2019). Historically, ncRNA was considered to be junk, or the by-products of transcription. However, recent studies have revealed that ncRNAs are crucial to many biological and pathological processes (Guttman et al., 2009; Ma et al., 2013; Bhan and Mandal, 2015; Yao et al., 2019). Non-coding RNAs are classified as either long non-coding RNAs or small non-coding RNAs (Manna and Sarkar, 2020), depending upon the number of nucleotides involved. LncRNAs are more than 200 nucleotides in length, and lack protein-coding capability (Chekanova, 2015; Yao et al., 2019). The mechanisms by which lncRNAs influence gene expression can be roughly divided into *cis-*acting and *trans-*acting (Figure 1). There are three possible mechanisms for *cis* action: (1) lncRNA recruits transcription factors to a locus to regulate the expression of a nearby gene (Figure 1A); (2) regulation of nearby genes during lncRNA transcription and shearing (Figure 1B); and (3) regulation of nearby genes by the lncRNA promoter or original DNA of the locus (Figure 1C; Ma et al., 2013; Kopp and Mendell, 2018; Yao et al., 2019). There are also three possible mechanisms of *trans* action: (1) the lncRNA regulates the chromatin state and gene transcription in distant regions (Figure 1D); (2) the lncRNA regulates the nuclear structure and organization (Figure 1E); and (3) the lncRNA binds to one or more proteins or other RNAs to regulate their activity (Figure 1F; Ma et al., 2013; Kopp and Mendell, 2018; Yao et al., 2019). LncRNAs contribute to a wide range of biological processes because of their diverse mechanisms of action (Chen and Shan, 2020).

Currently, about 2,000 RNA-binding proteins (RBPs) have been identified in humans (Corley et al., 2020; Qin et al., 2020). RBPs have been recognized as key regulators of transcription and post-transcriptional processing (Janakiraman et al., 2018; Xu et al., 2019). A large body of work has shown that many RBPs have impacts on tumorigenesis and cancer cell survival (Gao et al., 2020; Schultz et al., 2020; Weiße et al., 2020). Recent research into ncRNAs has revealed the existence of complex RBP-ncRNA interactions (Jonas et al., 2020). Mechanistically, RBPs regulate RNA splicing, polyadenylation, mRNA stability, mRNA localization, and mRNA translation, by interacting with coding and non-coding RNAs and other proteins (Qin et al., 2020; Zang et al., 2020). LncRNAs can also affect the stability of RBPs (Zhang et al., 2020). The lncRNA PVT1 has no effect on the stability of MYC mRNA, but it can protect MYC protein from phosphorylation-mediated degradation in 8q24 amplified human cancer cells (Ferreri et al., 2010). The lncRNA GAS5 interacts with the WW domain of the YAP protein in colorectal cancer, and promotes the degradation of Yes1-related transcriptional regulators (YAP) through the ubiquitin-proteasome pathway (Ni et al., 2019).

UPF1 is a common RNA-binding protein, and is a critical molecule to the RNA decay pathway. The most common RNA decay pathways in which UPF1 participates are nonsense-mediated mRNA decay (NMD) (Figure 2A) and staufen (STAU)-mediated mRNA decay (SMD) (Figure 2B) (Kim and Maquat, 2019). UPF1 plays a role in many other pathways, such as replication-dependent histone mRNA decay (HMD), glucocorticoid receptor-mediated mRNA decay (GMD), regnase 1-mediated mRNA decay (RMD), and tudor-staphylococcal/micrococcal-like nuclease (TSN)-mediated microRNA decay (TumiD) (Kim and Maquat, 2019). In NMD, UPF1 binds to a premature termination codon (PTC) though translation termination complex, which consists of eRF1, eRF3, SMG1, SMG8, and SMG9 (Kurosaki and Maquat, 2016). PTCs are located more than 50 to 55 nucleotides upstream of an exon–exon junction, and exon junction complexes (EJCs) are 20 to 24 nucleotides upstream of an exon–exon junction (Hug et al., 2016; Kurosaki and Maquat, 2016). EJCs can be removed by ribosomes. However, the process will be stopped when there is a PTC (Kurosaki and Maquat, 2016). UPF2, together with UPF3 or UPF3X, binds to EJCs (Popp and Maquat, 2018). UPF1 then interacts with UPF2 and triggers the NMD pathway (Kurosaki and Maquat, 2016; Popp and Maquat, 2018; Kim and Maquat, 2019). UPF1 can mediate the degradation of NMD substrates through the phosphorylation/dephosphorylation cycle (Schoenberg and Maquat, 2012). NMD identifies and degrades mRNA which have PTCs, so that the synthesis of truncated proteins is prevented (Fatscher et al., 2015; Li et al., 2017; Kim and Maquat, 2019). Recent studies have shown that NMD not only degrades abnormal transcription products, but also regulates normal gene expression (Kurosaki et al., 2014; Kim and Maquat, 2019). The stability of 5–10% of normal physiological mRNAs are regulated by NMD (Kim and Maquat, 2019). The role of NMD in tumor development is complex. In some tumors, the expression of tumor suppressor genes is downregulated, because NMD selects specific mutations which cause destruction of tumor suppressor mRNAs, and tumor cells adjust their NMD activity to adapt to their microenvironment (Popp and Maquat, 2018). Mutations of the NMD machinery have been observed in tumors (Popp and Maquat, 2018). STAU1 binding sites (SBS) are a key trigger of SMD. There are two types of SBSs: one is a stem-loop structure formed by intramolecular base-paring within an mRNA 3′ UTR, and the other is formed by two Alu elements (Gong and Maquat, 2011; Park and Maquat, 2013). One of the two Alu elements comes from the mRNA of the SMD target, and the other Alu element comes from a lncRNA (Gong and Maquat, 2011). In SMD, STAU1 dimer binds to SBS and recruits and binds UPF2 together with UPF1, resulting in UPF1 phosphorylation and RNA degradation (Gong and Maquat, 2011; Plank and Wilkinson, 2018; Gowravaram et al., 2019). The SMD and NMD pathways share the steps of UPF1 phosphorylation. STAU1 and another NMD factor, UPF2, bind the overlapping region of the UPF1 CH domain, so that SMD and NMD are competing pathways (Park and Maquat, 2013).

In this review, we primarily discuss dysregulated lncRNAs in tumors, and the way in which these lncRNAs interact with UPF1 to regulate gene expression in tumors (Table 1).

**TABLE 1:** LncRNAs mentioned in the review.

lncRNA	Tumor	Expression	Mechanism	References
**ZFPM2-AS1**	Lung cancer	up-regulated	The synergistic effect of ZFPM2-AS1 and UPF1 destroys the stability of ZFPM2 mRNA	Han et al., 2020
**MACC1-AS1**	Non-small cell lung cancer	up-regulated	The combination of LncRNA MACC1-AS1 and UPF1 weakens the stability of LATS1/2 mRNA.	Wang et al., 2020
**MALAT1**	Gastric cancer	up-regulated	UPF1 binds to MALAT1 and promotes the degradation of MALAT1	Li et al., 2017
**NR4A1AS**	Colorectal cancer	up-regulated	NR4A1AS can competitively bind to NR4A1 mRNA with UPF1 to maintain the stability of NR4A1 mRNA	Xie et al., 2019
**SNHG6**	Colorectal cancer	up-regulated	SNHG6 binds UPF1 and up-regulates the expression of UPF1 then destroys the stability of smad7 mRNA	Wang et al., 2019
	Hepatocellular carcinoma	up-regulated		Chang et al., 2016a,b
**TINCR**	Gastric cancer	up-regulated	TINCR can bind to STAU1and UPF1 and degrade KLF2 mRNA	Xu et al., 2015
**UCA1**	Hepatocellular carcinoma	up-regulated	UPF1 binds to UCA1and reduces the expression of UCA1	Zhou et al., 2019
**SNAI3-AS1**	Hepatocellular carcinoma	up-regulated	SNAI3-AS1 binds UPF1 and up-regulates the expression of UPF1, then destroys the stability of smad7 mRNA	Li Y et al., 2019
**Linc00313**	Glioma	up-regulated	The combination of UPF1 and Linc00313 delays the degradation of Linc00313	Shao et al., 2019
**FBXL19-AS1**	Glioma endothelial cells	up-regulated	BXL19-AS1 can bind to UPF1 and down-regulate the expression of ZNF765 mRNA through the SMD pathway	Liu et al., 2020
**LINC00346**	Glioma endothelial cells	up-regulated	LINC00346 binds UPF1 and promotes the degradation of ZNF655 mRNA through the SMD pathway	Yang et al., 2020
**HCG15**	Glioma	up-regulated	HCG15 binds UPF1 and promotes the degradation of ZNF331 mRNA through the SMD pathway	Jing et al., 2020
**SNHG20**	Glioma	up-regulated	SNHG20 binds UPF1 and promotes the degradation of FOXK1 mRNA through the SMD pathway	Li X et al., 2019
**BDNF-AS**	Glioma	down-regulated	BDNF-AS binds UPF1 and promotes the degradation of RAX2mRNA through the SMD pathway	Su et al., 2020

## Mechanisms of lncRNA Interactions With UPF1

**FIGURE 3:**
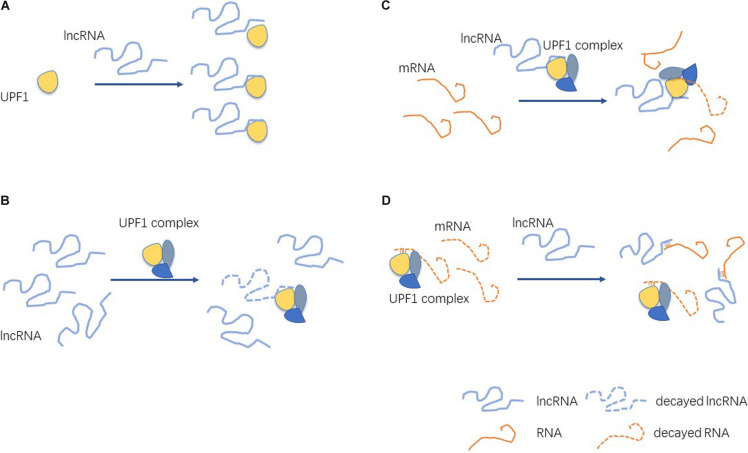
Mechanisms of lncRNA interactions with UPF1. **(A)** lncRNAs bind UPF1 and affect the expression of UPF1. **(B)** lncRNAs bind UPF1 and affect the stability of mRNA. **(C)** UPF1 binds lncRNAs and affects the expression of lncRNAs. **(D)** UPF1 and lncRNA competitively bind mRNA.

In order to understand the nature and effects of the interactions between lncRNA and UPF1, we can divide their mechanisms of action into the following four categories (Figure 3).

### LncRNAs Bind UPF1 and Affect the Expression of UPF1

SNHG6 is upregulated in hepatocellular carcinoma (HCC) tissues and cell lines, in which it promotes the proliferation, invasion and migration of hepatocellular carcinoma cells, inhibits apoptosis, and induces cell cycle (Chang et al., 2016b). UPF1 is upregulated when SNHG6 is knocked down in human hepatocellular carcinoma cell lines (Chang et al., 2016b). SNHG6 is also upregulated in colorectal cancer (CRC) tissues and cell lines (Wang et al., 2019). The expression of UPF1 has an inverse correlation with SNHG6 in CRC tissues (Wang et al., 2019); when SNHG6 is knocked down, the expression of UPF1 is upregulated (Wang et al., 2019). These results indicate that SNHG6 influences the expression of UPF1. The lncRNA SNAI3-AS1 is upregulated in HCC tissues and cells (Li Y et al., 2019). Knockdown of SNAI3-AS1 can inhibit HCC cell invasion by upregulating the expression of UPF1 (Li Y et al., 2019). SNAI3-AS1 therefore reversely regulates the expression of UPF1 (Li Y et al., 2019).

### LncRNAs Bind UPF1 and Affect the Stability of mRNA

The lncRNA ZFPM2-AS1 is upregulated in lung adenocarcinoma tissues and cell lines, promoting the invasion and epithelial-mesenchymal transition (EMT) of lung adenocarcinoma cells (Han et al., 2020). ZFPM2, a gene located close to ZFPM2-AS1, is negatively regulated by LncRNA ZFPM2-AS1, and downregulated in lung adenocarcinoma tissues (Han et al., 2020). UPF1 participates in the regulation of ZFPM2-AS1 by ZFPM2. UPF1 can bind to the 3′ UTR region of ZFPM2-AS1 and ZFPM2 mRNA, and forms a binding complex in lung adenocarcinoma cells (Han et al., 2020). The synergistic effect of ZFPM2-AS1 and UPF1 destroys the stability of ZFPM2 mRNA, down-regulates the expression of ZFPM2, and promotes the proliferation, invasion, and EMT of lung adenocarcinoma cells (Han et al., 2020). The lncRNA MACC1-AS1 positively regulates the stemness of non-small cell lung cancer cells by downregulating the expression of LATS1/2, the key factor of the HIPPO pathway, to activate the HIPPO pathway (Wang et al., 2020). Both the lncRNA MACC1-AS1 and LATS1/2 mRNA can bind to UPF1 (Wang et al., 2020). Silencing UPF1 can attenuate the downregulation of LATS1/2 by overexpression of MACC1-AS1 (Wang et al., 2020). The combination of MACC1-AS1 and UPF1 therefore weakens the stability of LATS1/2 mRNA (Wang et al., 2020). As previously mentioned, upregulation of SNHG6 reduces the expression of UPF1 in hepatocellular carcinoma and colorectal cancer (Chang et al., 2016b; Wang et al., 2019). Upregulated SNAI3-AS1 reversely regulates the expression of UPF1 in hepatocellular carcinoma (Li Y et al., 2019). Further studies have shown that SNHG6 and SNAI3-AS1 bind to UPF1 and promote the decay of smad7 mRNA (Chang et al., 2016a,b; Li Y et al., 2019). Smad7 is a negative regulator of the TGF-β/SMAD pathway (Lukas et al., 2017). When Smad7 is downregulated, the TGF-(β/SMAD pathway becomes active, and induces the EMT (Colak and Ten, 2017; Lukas et al., 2017). It has been reported that the expression of KLF2 protein is lower in gastric tumors, and KLF2 overexpression markedly enhanced cell apoptosis, and induced cell cycle arrest (Wang et al., 2017). The lncRNA TINCR is strongly upregulated in gastric cancer, and promotes the growth of gastric cancer cells by regulating the expression of KLF2 (Xu et al., 2015). RNA Immunoprecipitation (RIP) experiments confirmed that TINCR can bind to STAU1, and RNA pull-down assays revealed TINCR also binds to UPF1 (Xu et al., 2015). Further study confirmed that KLF2 mRNA, a target of SMD, was enriched by STAU1 antibody (Xu et al., 2015). STAU1 also binds to the 3′ UTR of KLF2 mRNA, and the KLF2 mRNA half-life was increased following the downregulation of STAU1 or TINCR (Xu et al., 2015). These results suggest that TINCR affects the expression of KLF2 through the SMD pathway (Xu et al., 2015). As a key factor of SMD, UPF1 interacts with a variety of lncRNAs, and plays an important role in tumorigenesis and the progression of glioma. The blood-tumor barrier (BTB) attenuates the efficacy of chemotherapy for glioma. The lncRNA FBXL19-AS1 is overexpressed in the cytoplasm of glioma microvessels and glioma endothelial cells (GECs) (Liu et al., 2020), and knockdown of FBXL19-AS1 increases the permeability of the BTB (Liu et al., 2020). Low expression of zinc finger protein 765 (ZNF765) in GEC improves the permeability of the BTB by inhibiting the promoter activity of tight junction-related proteins. Further studies have found that the binding of BXL19-AS1 and the 3′ UTR of ZNF765 mRNA produces an SBS (Liu et al., 2020). The half-life of ZNF765 mRNA was reduced when BXL19-AS1 was overexpressed, STAU1 was knocked down, or UPF1 was knocked down. BXL19-AS1 therefore affects the expression of ZNF765 mRNA through the SMD pathway (Liu et al., 2020). In GEC, LINC00346 is significantly increased, and the zinc finger protein 655 (ZNF655) is decreased (Yang et al., 2020). LINC00346 inhibition or ZNF655 overexpression hinder GEC angiogenesis (Yang et al., 2020). In terms of mechanism, LINC00346 and the 3′ UTR of ZNF655 mRNA make up the SBS (Yang et al., 2020). RIP experiments showed that LINC00345 and ZNF655 mRNA both bind to STAU1 (Yang et al., 2020). When LINC00345, STAU1, or UPF1 was silenced, the half-life of ZNF655 mRNA was prolonged in GECs (Yang et al., 2020). LINC00345 therefore promotes the degradation of ZNF655 mRNA through SMD to promote angiogenesis in glioma (Yang et al., 2020). Similarly, the lncRNA HCG15 is highly expressed in gliomas, and promotes the degradation of ZNF331 mRNA through the SMD pathway. The lncRNA SNHG20 promotes the degradation of FOXK1 mRNA through the SMD pathway, thereby promoting the formation of glioma vascular mimicry and playing a role in promoting cancer (Li X et al., 2019; Jing et al., 2020). The down-regulation of the lncRNA BDNF-AS can promote the degradation of RAX2 mRNA via the SMD pathway, and inhibit the progression of malignancy in glioblastoma cells (Su et al., 2020).

### UPF1 Binds LncRNA and Affects the Expression of LncRNA

MALAT1 is one of the most studied lncRNAs (Ji et al., 2003). MALAT1 is upregulated in a variety of tumors, including gastric cancer (Sun and Ma, 2019). MALAT1 promotes the proliferation, migration, invasion, and autophagy of gastric cancer by regulating the expression level of miRNAs (Malakar et al., 2017; YiRen et al., 2017; Li H et al., 2019; Lu et al., 2019). However, the expression of MALAT1 is regulated by UPF1. The expression of UPF1 is negatively correlated with the expression of MALAT1 in gastric cancer (Li et al., 2017). When UPF1 is overexpressed, the expression of MALAT1 is downregulated, and the half-life of MALAT1 is shortened (Li et al., 2017). UPF1 binds to MALAT1 and promotes its degradation, thereby inhibiting the expression of MALAT1 in gastric cancer (Li et al., 2017). In HCC, the expression of UPF1 is decreased, and silencing of UPF1 promotes the growth and invasion of HCC cells (Zhou et al., 2019). Knockdown of UPF1 in HCC cells increases the expression of UCA1 (Zhou et al., 2019). RIP experiments confirmed the combination of UPF1 (Zhou et al., 2019). The stability of UCA1 RNA was tested in HCC cells with UPF1 knocked down, and the decay rate of UCA1 was seen to increase (Zhou et al., 2019). Knockdown of UCA1 ameliorated the effect of UPF1 knockdown on HCC growth and invasion, indicating that UCA1 mediates the effect of UPF1 on the invasion and proliferation of hepatocellular carcinoma cells (Zhou et al., 2019). UPF1 and Linc00313 are both upregulated in glioma tissues and cells. Knocking down the expression of Linc00313 or UPF1 can inhibit the proliferation, invasion and migration of glioma cells, and promote apoptosis (Shao et al., 2019). RIP results revealed the combination of UPF1 and Linc00313 (Shao et al., 2019). When UPF1 is overexpressed, the half-life of Linc00313 is increased, and when UPF1 is knocked down, the opposite result was found (Shao et al., 2019). UPF1 appears to delay the degradation of Linc00313 and enhance the effect of Linc00313 on glioma (Shao et al., 2019).

### UPF1 and LncRNA Competitively Bind mRNA

The upregulation of lncRNA NR4A1AS expression in colorectal cancer tissue is positively correlated with the expression of NR4A1 mRNA (Xie et al., 2019). NR4A1AS increases the stability of NR4A1 mRNA by forming RNA duplexes in CRC cells, thereby regulating the expression of NR4A1 (Xie et al., 2019). NR4A1 mRNA can also bind to UPF1, to increase its mRNA degradation rate (Xie et al., 2019). However, NR4A1AS cannot bind to UPF1. After silencing NR4A1AS in colorectal cancer cells, the NR4A1 mRNA bound to UPF1 was seen to almost double (Xie et al., 2019). These results indicate that NR4A1AS can bind to NR4A1 mRNA in competition with UPF1, to maintain the stability of NR4A1 mRNA in CRC cells and regulate the expression of NR4A1.

## Discussion

A considerable body of research shows that lncRNA is widely involved in tumorigenesis and the progression of various cancers. LncRNA regulates the expression and functions of other genes by binding to DNA, RNAs and proteins, and therefore participates in producing the tumor phenotype (Ramnarine et al., 2019; Cheng et al., 2020; Huang et al., 2020). As an RNA binding protein with multiple identities, UPF1 can participate in both the NMD pathway and the SMD pathway to regulate RNA stability and maintain homeostasis (Kim et al., 2007; Nasif et al., 2018). After UPF1 binds to lncRNA, the stability of the lncRNAs decreases in most cases. However, one study indicated that UPF1 binds to lncRNA and increases the stability of lncRNAs. These conflicting results seem to indicate a dual role for UPF1. The interactions between lncRNAs and UPF1 can regulate the degradation rate of other mRNAs. Antisense lncRNA may competitively bind to UPF1 to regulate mRNA expression. In some cases, the binding of lncRNA and UPF1 will affect the expression of UPF1, but the specific mechanism involved needs further exploration. Some studies have shown that after lncRNAs bind to RBPs, the stability of the RBPs is affected. LncRNA FAM83H-AS1 can bind to the HuR protein and promote its stability. However, the lncRNA MEG3 binds to the p-STAT3 protein, and promotes its degradation through ubiquitination (Dou et al., 2019; Zhang and Gao, 2019). Therefore, it appears that the binding of lncRNA and UPF1 may affect the stability of UPF1. Further exploration of the interactions and mechanisms underlying the relationship between lncRNA and UPF1 will help us to understand the influence of lncRNA and RBP on pathophysiological processes.

## Author Contributions

JH and XM contributed to the conception of the study, discussed, and improved the revised manuscript. JH wrote the manuscript. Both authors read and approved the final manuscript.

## Conflict of Interest

The authors declare that the research was conducted in the absence of any commercial or financial relationships that could be construed as a potential conflict of interest.
